# Relation among hypertriglyceridaemia, cardiometabolic disease, and hereditary factors—design and rationale of the Stockholm hyperTRIglyceridaemia REGister study

**DOI:** 10.1093/ehjopen/oeae010

**Published:** 2024-02-20

**Authors:** Daniel P Andersson, Karin Littmann, Gustav Kindborg, Daniel Eklund, Kristina Sejersen, Jane Yan, Daniel Eriksson Hogling, Paolo Parini, Jonas Brinck

**Affiliations:** Department of Medicine Huddinge, Karolinska Institutet, Cardio Metabolic Unit, C2:94, Karolinska University Hospital Huddinge, 141 86 Stockholm, Sweden; Medical Unit Endocrinology, C2:94, Karolinska University Hospital Huddinge, 141 86 Stockholm, Sweden; Department of Medicine Huddinge, Karolinska Institutet, Cardio Metabolic Unit, C2:94, Karolinska University Hospital Huddinge, 141 86 Stockholm, Sweden; Medical Unit Endocrinology, C2:94, Karolinska University Hospital Huddinge, 141 86 Stockholm, Sweden; Department of Medicine Huddinge, Karolinska Institutet, Cardio Metabolic Unit, C2:94, Karolinska University Hospital Huddinge, 141 86 Stockholm, Sweden; Medical Unit Endocrinology, C2:94, Karolinska University Hospital Huddinge, 141 86 Stockholm, Sweden; Medical Unit Clinical Chemistry, C1-62, Karolinska University Laboratory, 141 86 Stockholm, Sweden; Department of Medical Sciences, Section of Clinical Chemistry, Uppsala University, Uppsala University Hospital, 751 85 Uppsala, Sweden; Unilabs AB, Unilabs Laboratory Medicine Stockholm, Section of Clinical Chemistry, 171 54 Solna, Sweden; Institute of Environmental Medicine, Unit of Biostatistics, Karolinska Institutet, Nobels väg 13, 17 177 Stockholm, Sweden; Department of Medicine Huddinge, Karolinska Institutet, Cardio Metabolic Unit, C2:94, Karolinska University Hospital Huddinge, 141 86 Stockholm, Sweden; Medical Unit Endocrinology, C2:94, Karolinska University Hospital Huddinge, 141 86 Stockholm, Sweden; Department of Medicine Huddinge, Karolinska Institutet, Cardio Metabolic Unit, C2:94, Karolinska University Hospital Huddinge, 141 86 Stockholm, Sweden; Medical Unit Endocrinology, C2:94, Karolinska University Hospital Huddinge, 141 86 Stockholm, Sweden; Department of Laboratory Medicine, Cardio Metabolic Unit, Karolinska Institutet, Alfred Nobels Allé 8, 141 52 Huddinge, Sweden; Department of Medicine Huddinge, Karolinska Institutet, Cardio Metabolic Unit, C2:94, Karolinska University Hospital Huddinge, 141 86 Stockholm, Sweden; Medical Unit Endocrinology, C2:94, Karolinska University Hospital Huddinge, 141 86 Stockholm, Sweden

**Keywords:** Hypertriglyceridaemia, Dyslipidaemia, Cardiovascular disease, Cardiometabolic disease, Pancreatitis

## Abstract

**Aims:**

Hypertriglyceridaemia (hTG) is associated with atherosclerotic cardiovascular disease, pancreatitis, and non-alcoholic fatty liver disease (NAFLD) in large population-based studies. The understanding of the impact of hereditary hTG and cardiometabolic disease status on the development of hTG and its associated cardiometabolic outcomes is more limited. We aimed to establish a multigenerational cohort to enable studies of the relationship between hTG, cardiometabolic disease and hereditary factors.

**Methods and results:**

The population-based observational Stockholm hyperTRIglyceridaemia REGister (STRIREG) study includes 1 460 184 index individuals who have measured plasma triglycerides in the clinical routine in Region Stockholm, Sweden, between 1 January 2000 and 31 December 2021. The laboratory measurements also included basic haematology, blood lipid panel, liver function tests, and HbA1c. Using the Swedish Multi-Generation register, 2 147 635 parents and siblings to the indexes were identified to form the complete study cohort. Laboratory data from participants were combined with data from several national registers that provided information on the cause of death, medical diagnoses, dispensed medicines, and socioeconomic factors including country of birth, education level, and marital status.

**Conclusion:**

The multi-generational longitudinal STRIREG cohort provides a unique opportunity to investigate different aspects of hTG as well as heredity for other metabolic diseases. Important outcome measures include mortality, cardiovascular mortality, major cardiovascular events, development of incident diabetes, and NAFLD. The STRIREG study will provide a deeper understanding of the impact of hereditary factors and associated cardiometabolic complications.

## Graphical Abstract

**Graphical Abstract:**
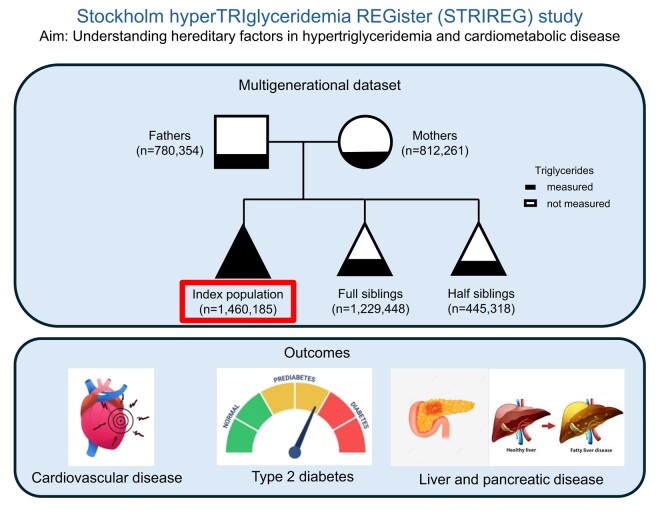

## Introduction

Hypertriglyceridaemia (hTG), defined as fasting plasma triglycerides >1.7 mmol/L (>150 mg/dL),^1^ is a risk factor for atherosclerotic cardiovascular disease (ASCVD),^2^ pancreatitis,^3^ and non-alcoholic fatty liver disease (NAFLD).^4^ According to the European Atherosclerosis Society’s definition of hTG, moderately (1.7–5.7 mmol/L or 150–500 mg/dL) to severely (5.7–10.0 mmol/L or 500–880 mg/dL) elevated triglycerides (Tg) is common in the non-fasting state and occurs transiently in ∼10% of the adult population, whereas extreme (>10.0 mmol/L or >880 mg/dL) triglyceride levels are rare (0.1–0.2%).^1,5^ The aetiology of hTG is in most cases multi-factorial. Studies of the genetic architecture of hTG have revealed that monogenetic forms of hTG, e.g. familial hyperchylomicronaemia syndrome, are extremely rare.^6^ In contrast, frequently observed mild to moderately elevated states of hTG are multi-genic in nature and result from a cumulative burden of common and rare variants in many genes and can be quantified by genetic risk scores.^7^ The presence of both heterozygous pathogenic variants and a high polygenetic risk score increases the risk for severe hTG.^8^ The genetic influence on the plasma lipoprotein levels and the lipoprotein lipidome is highly variable and has been estimated to be 20–50%.^9^ In the clinical setting, hTG is most often caused by the cumulative effect of multiple genetic risk variants and is exacerbated by lifestyle factors, metabolic disease, and side effects of medications.^10^

Patients with hTG remain in the epicentre for the development of cardiovascular disease despite recent decades of intensified dyslipidaemia treatment.^2,11–14^ A Tg level >1.7 mmol/L (>150 mg/dL) constitutes residual cardiovascular risk for patients in secondary prevention on effective LDL cholesterol lowering therapy^15^ and in individuals with subclinical atherosclerosis regardless of their LDL cholesterol level.^16^ However, strategies to pharmacologically lower plasma Tg levels to reduce ASCVD events have not been straightforward. In the era when statins form the basis for dyslipidaemia treatment to reduce cardiovascular risk, the prospective outcome trials with peroxisome proliferator-activated receptors alfa agonist (i.e. fibrates) or niacin to reduce hTG have failed to show benefit.^17–21^ The only successful outcome trial targeting individuals with hTG in the prevention of cardiovascular outcomes could not show an association between the reduction of hTG and the reduction of cardiovascular events.^22^ New mechanistic approaches in pharmacological treatment for hTG are under development, including monoclonal antibodies, anti-sense oligonucleotides, and small interfering RNAs.^23^ The anti-sense oligonucleotide volanesorsen directed against apoC3, a potent inhibitor of lipoprotein lipase, has been shown to be very efficient in reducing plasma triglyceride levels in patients with familial chylomicronaemia syndrome and with severe hTG.^24,25^ Furthermore, angiopoietin-like (ANGPTL) protein family, including ANGPTL 3, 4, and 8, is another important target through their function as regulators of intravascular lipolysis of triglyceride-rich lipoproteins.^26^ The ANGPTL3 protein inhibits lipoprotein lipase and hepatic lipase, and the monoclonal antibody evinacumab^27^ and the anti-sense oligonucleotide vupanorsen^28^ have been shown to have strong plasma triglyceride-lowering effects. However, due to their high cost and sparse availability, the selection of patients who benefit the most from treatment is crucial.

The aim of the Stockholm hyperTRIglyceridaemia REGister (STRIREG) study is to increase the knowledge of hTG and its association with ASCVD outcomes and cardiometabolic comorbidities, with a specific focus on the effects of hereditary hTG. In the STRIREG studies, hereditary hTG is defined as when an index has a first-degree relative with hTG, thus serving as a biochemical surrogate for genetic inheritance. The study results may be useful in risk stratification in modern hTG treatment. The STRIREG cohort includes more than 1.4 million individuals distributed across three generations with a Tg measurement and an additional 2.1 million individuals associated with first-degree relatives, with an observational period of up to 21 years.

## Methods

### Ethics

This observational cohort study was approved by the Swedish Ethical Review Authority (Dnr 2021-03883) and conducted according to the principles of the Helsinki Declaration.

### Study cohort and baseline

**Figure 1:**
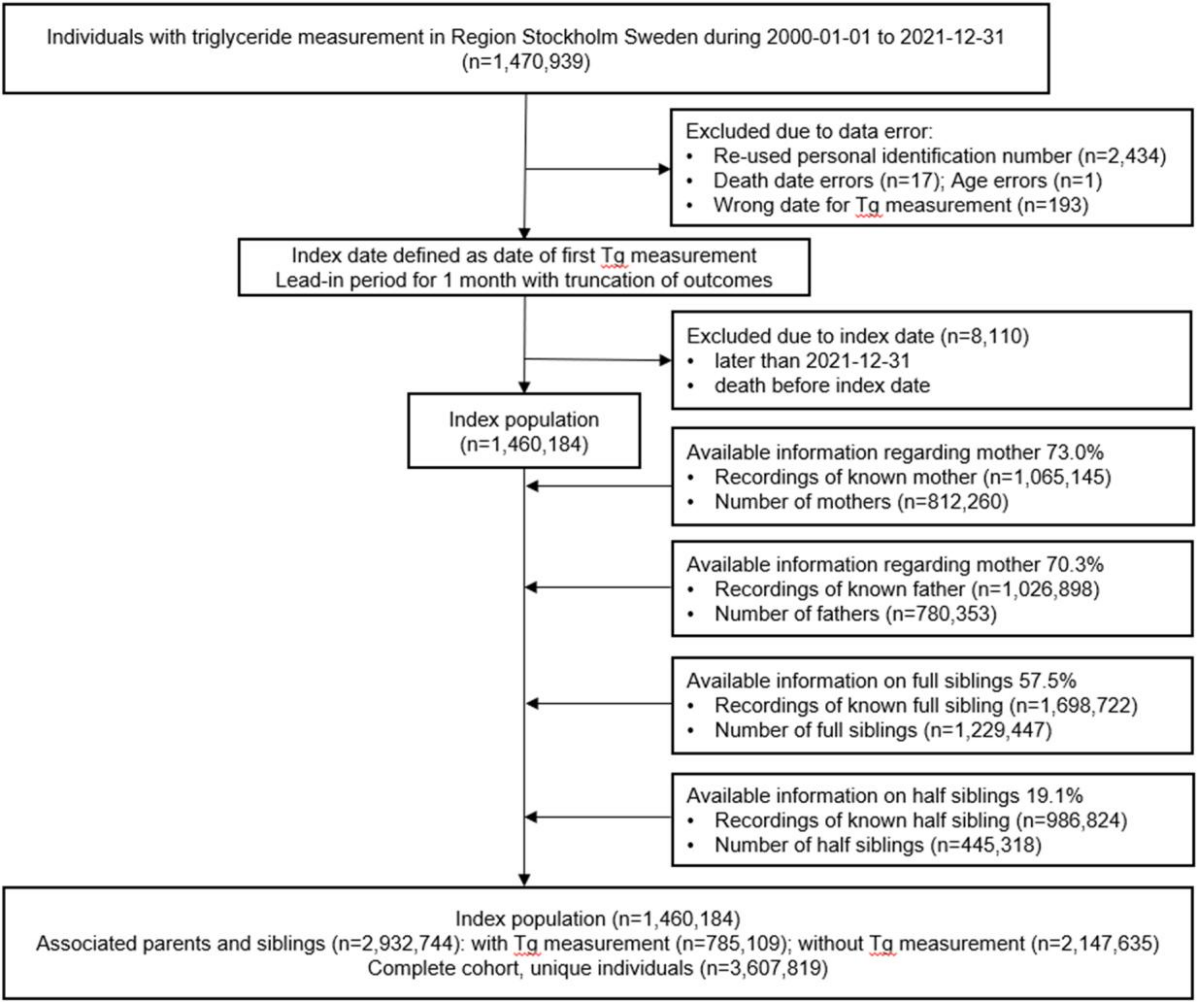
The flowchart of the inclusion of participants in the Stockholm hyperTRIglyceridaemia REGister cohort. Individuals with a Swedish personal identification number who had been sampled for plasma triglycerides in clinical routine at the Karolinska University Laboratory or at Unilabs AB Laboratory in Region Stockholm between 1 January 2000 and 31 December 2021 constituted the index population. Parents and siblings to indexes were associated with the cohort by interlinking personal identification numbers in the Multi-Generation register, Statistics Sweden, to form the complete Stockholm hyperTRIglyceridaemia REGister cohort.

The STRIREG study is a retrospective longitudinal general population–based register study including all individuals who had had at least one plasma Tg measurement between 1 January 2000 and 31 December 2021 at Karolinska University Laboratory or Unilabs AB in Region Stockholm (population 2.41 million in 2021). The exclusion criteria were a lack of a unique Swedish personal identification number (PIN). The index population consisted of 1 460 184 individuals between the ages of 0 and 107 years. The index population was extended to form the complete cohort (*n* = 3 607 819) by associating the parents and the siblings (*n* = 2 147 635) to the indexes by interlinkage of PINs via the Multi-Generation register (see below). See *Figure 1* for the study flowchart of the inclusion of participants in the STRIREG cohort. The study baseline for the index population was defined as the date of participant’s first Tg measurement. A 1-month lead-in period with truncation of outcomes was used to avoid the bias of performing statistical analyses based on a Tg measurement sampled in a potential non-related acute clinical condition.

### Laboratory parameters

**Figure 2:**
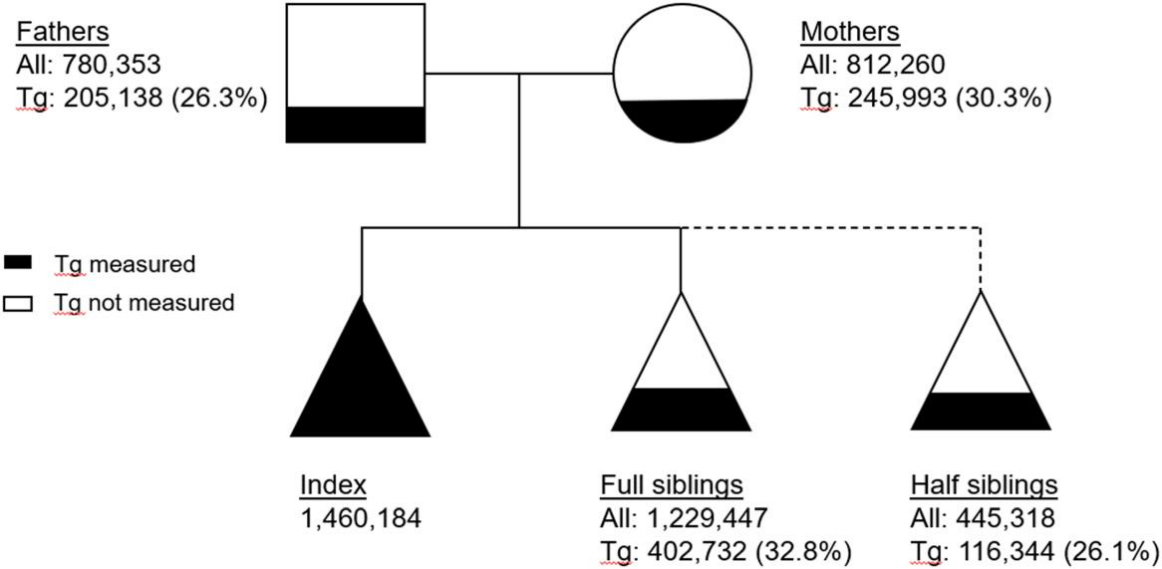
The number of study participants, their inter-individual relations, and the proportions with a triglyceride measurement in the Stockholm hyperTRIglyceridaemia REGister cohort. The Stockholm hyperTRIglyceridaemia REGister cohort has 3 607 819 unique participants of which 1 460 184 belong to the index population with a plasma triglyceride measurement. A participant can be an index, parent, and/or sibling, and parents and siblings can be related to several indexes but are counted only once. The filling of geometric figures reflects the proportion of participants in each subpopulation with a triglyceride measurement.

All laboratory measurements included in the STRIREG study were analysed at the two major clinical chemistry laboratories (Karolinska University Laboratory and Unilabs AB) in Region Stockholm, Sweden, using certified assays; the dataset does not contain genetic data. All Tg measurements were from the clinical routine and could be sampled in a fasting or non-fasting state. In total, the individuals in the index cohort had 6 481 744 plasma Tg measurements with a median of 2 measures per individual (inter-quartile range 1–5). In addition to plasma Tg, the following whole blood, plasma, or serum laboratory parameters for the index population were also included in the dataset: haematology (haemoglobin, red blood cell count, white blood cell count, and thrombocyte count), lipid panel [total cholesterol, HDL cholesterol, LDL cholesterol, apolipoprotein A1, apolipoprotein B, and lipoprotein(a)], kidney function test (creatinine and estimated glomerular filtration rate), haemoglobin A1c, liver function test (alanine transaminase, aspartate aminotransferase, albumin, alkaline phosphatase, and prothrombin time-international normalized ratio), amylase, pancreas amylase, alcohol consumption (phosphatidyl ethanol and carbohydrate-deficient transferrin), and thyroid function [thyroid-stimulating hormone, triiodothyronine (T3), and thyroxine (T4)]. The number and the proportion of individuals with a Tg measurement and their inter-individual relations in the complete STRIREG cohort are depicted in *Figure 2*.

### Data sources

Data from the following national Swedish registers were used to generate the complete data-set by the use of the Swedish PIN: The *Multi-Generation Register* (Statistics Sweden) provided the identities of the parents and the siblings to the index population. The *Longitudinal Integrated Database for Health Insurance and Labour Market Studies Register* (Statistics Sweden) provided data on socioeconomic factors including country of origin, educational level, annual income, and marital status. The *Cause of Death Register*, the *National Patient Register*, and the *Register for Prescribed and Dispensed Drugs* (National Board of Health and Welfare) provided data on the cause of death, provided data according to the International Classification of Diseases (ICD) codes (ICD9 and ICD10) from inpatient clinics (from 1987) and specialized outpatient clinics (from 2001), and provided data on dispensed drugs according to Anatomical Therapeutic Chemical Classification codes (from 2006), respectively. The representation of study participants from the index population and the associated parents’ and siblings’ cohorts in various data registers is presented in *Table 1*.

**Table 1:** Register data available for the Stockholm hyperTRIglyceridaemia REGister cohort

	Index (*N* = 1 460 184)	Associated parents and siblings (*N* = 2 932 744)	Total cohort (*N* = 3 607 819)
Female sex, *n* (%)	712 455 (48.8)	1 454 607 (49.6)	1 768 871 (49.0)
Age in years at the first Tg measurement, mean (SD)	49.1 (17.4)	Not applicable	Not applicable
Death register 1987–2022, *n* (%)	226 509 (15.5)	823 187 (28.1)	899 342 (24.9)
Cardiovascular death^a^ (ICD9: 401–440; ICD10: I10–79), *n* (%)	121 450 (8.3)	458 777 (15.6)	499 376 (13.8)
Diagnosis register 1987–2022, *n* (%)	1 037 968 (71.1)	1 903 887 (64.9)	2 345 340 (65.0)
Cancer (ICD9: 140–208; ICD10: C00–97), *n* (%)	289 097 (19.8)	611 764 (20.9)	710 583 (19.7)
Cardiovascular disease (ICD9: 401–440; ICD10: I10–79), *n* (%)	300 129 (20.6)	710 241 (24.2)	811 237 (22.5)
Chronic kidney disease (ICD10: N18–19), *n* (%)	64 397 (4.4)	96 515 (3.3)	118 492 (3.3)
Diabetes (ICD9: 250; ICD10: E10–E14), *n* (%)	174 049 (11.9)	285 305 (9.7)	351 473 (9.7)
Lipid disorders (ICD9: 272; ICD10: E78), *n* (%)	147 972 (10.1)	220 087 (7.5)	269 751 (7.5)
Dispensed drug register 2006–22 according to ATC, *n* (%)	1 186 345 (81.2)	675 756 (23.0)	1 188 900 (33.0)
Anticoagulants (B01A), *n* (%)	577 441 (39.5)	366 557 (12.5)	578 628 (16.0)
Cardiovascular disease (C01-03, C07-09), *n* (%)	816 013 (55.9)	500 558 (17.1)	817 738 (22.7)
Diabetes (A10), *n* (%)	205 898 (14.1)	121 730 (4.2)	206 175 (5.7)
Lipid lowering (C10), *n* (%)	469 569 (32.2)	298 164 (10.2)	470 164 (13.0)
Socioeconomic factors register 1990–2022, *n* (%)	1 438 538 (98.5)	2 352 456 (80.2)	3 010 322 (83.4)
Place of birth			
Sweden, *n* (%)	1 121 320 (76.8)	2 600 566 (88.7)	3 063 745 (84.9)
Europe, *n* (%)	163 263 (11.2)	191 658 (6.5)	283 674 (7.9)
Rest of the world, *n* (%)	175 602 (12.0)	140 523 (4.8)	260 402 (7.2)
Education			
Elementary school, *n* (%)	166 745 (11.4)	920 025 (31.4)	999 309 (27.7)
Secondary school, *n* (%)	448 692 (30.7)	809 820 (27.6)	985 645 (27.3)
Collage, *n* (%)	844 748 (57.9)	1 202 902 (41.0)	1 622 867 (45.0)

ATC, Anatomical Therapeutic Chemical classification system; SD, standard deviation.

^a^Includes ICD codes as underlying diagnosis and up to five contributory causes for fatal outcomes.

### Exposures and outcomes

**Figure 3:**
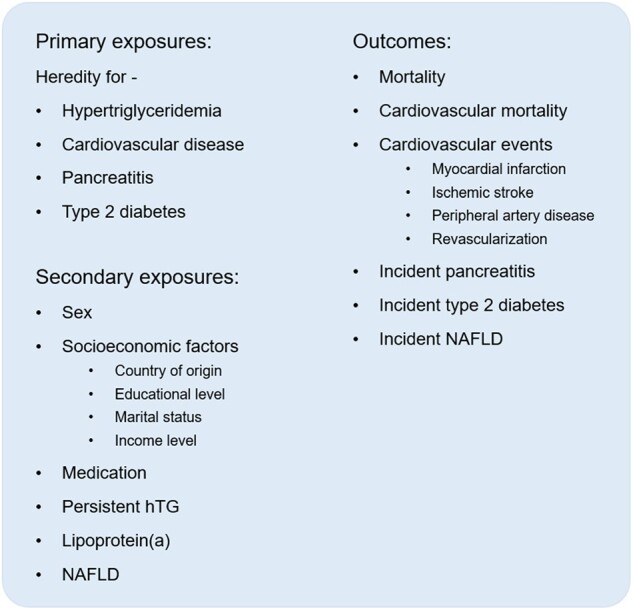
The exposures and outcomes are investigated in the Stockholm hyperTRIglyceridaemia REGister study. The Stockholm hyperTRIglyceridaemia REGister cohort, containing participants with triglyceride measurement from up to three generations, focuses on the role of hereditary hypertriglyceridaemia in the development of cardiometabolic disease. NAFLD, non-alcoholic fatty liver disease.

The objective of the STRIREG study is to increase the understanding of hTG and its association with ASCVD outcomes and cardiometabolic comorbidities with a focus on hereditary factors. The study exposures and outcomes that are being investigated are listed in *Figure 3*. The study is registered at ClinicalTrials.gov (NCT06104943).

### Data management and statistical analyses

Data management and statistical analyses will be handled at the Department of Medicine (MEDH7), Unit of Endocrinology, and the Institute of Environmental Medicine, Unit of Biostatistics, Karolinska Institutet, Stockholm. The statistical analysis will be performed using statistical software programmes (STATA version 16.1, StataCorp, College Station, TX, USA, and R version 4.1.2, R core team, Vienna, Austria). We will use unadjusted and multiple adjusted Cox proportional hazard regression models to study the association between hereditary factors in different strata of plasma triglycerides with different outcomes and in some additional analyses also inverse probability of treatment weighting or propensity score matching. To adjust for possible differences during the long inclusion period, a separate sensitivity analysis will be performed taking this into consideration. The identity of participants is coded into study-specific identification number (pseudonymization of the data), and data will be handled cautiously to avoid access by unauthorized persons. All data will be presented at the group level, so that no specific participant can be identified.

### Strengths and limitations

The major strengths of the STRIREG study are the cohort size and the linkage of first-degree relatives to indexes, thus enabling the study of the role of hereditary factors in cardiometabolic disease development. In addition, by using validated national Swedish registers for dispensed drugs and socioeconomic factors, we will be able to study a broad range of exposures and outcomes.

The study limitations are several. The longitudinal observational study design makes it impossible to infer causality. Although data have been registered prospectively, several different laboratory methods to measure plasma Tg have been used during the follow-up time of over two decades, and we cannot exclude a minor drift in measurement results over time. However, both participating laboratories are externally accredited and perform regular internal and external quality controls.^29^ In addition, they follow the national recommendation for reference limits and quality goals with an acceptable deviation at the upper limit of the reference interval (for plasma Tg, set to 0.39 mmol/L or 34 mg/dL).^30^ As in all observational studies, there may be residual confounding, not the least by confounding by indication; all Tg measurements were performed in the clinical routine, but no information is available on the indication of the sampling. Furthermore, although the Swedish national registers have good coverage and accuracy, there might still be data missing or incorrect data.^31^

## Discussion

The observational STRIREG study focuses on hTG and its importance for cardiometabolic disease. The unique Swedish Multi-Generation register enables us to investigate the impact of heredity for hTG, Type 2 diabetes, or ASCVD on the susceptibility for the development of ASCVD, Type 2 diabetes, pancreatitis, or NAFLD at different levels of hTG. In addition, the study will explore whether sex or socioeconomic factors affect the prevalence of hTG and cardiometabolic outcomes. The size and inclusiveness of the STRIREG cohort will allow for population-based results and generalizable conclusions.

It is complex to investigate and interpret the role of plasma triglycerides in the development of cardiometabolic disease. Triglycerides constitute the body’s principal energy source, and plasma triglycerides are thus also a reflection of the fluctuation in energy balance among dietary uptake, peripheral tissue expenditure, and adipose storage.^32^ The plasma triglyceride level is under regulation by hormones involving numerous metabolic pathways with the liver as the key control organ. The plasma triglyceride level is, on a granular level, determined by the particle numbers of the triglyceride-rich lipoproteins, their content, and their complex metabolism.^33^ The initial intervention step to reduce triglyceride levels and metabolic risk includes a healthy diet, reduced intake of alcohol, and physical exercise that can lead to a substantial reduction in plasma triglyceride levels.^34^ In addition, improved metabolic control of Type 2 diabetes achieved with glucagon-like peptide-1 receptor agonists (GLP-1 RAs) or a combination of glucose-dependent insulinotropic polypeptide and GLP-1 RA for weight reduction purposes also reduces plasma triglycerides.^35,36^

Inflammation is an important mediator in the pathophysiological process leading to atherosclerosis in dyslipidaemia.^37,38^ Stemming inflammatory signalling pathways in patients with manifest ASCVD can reduce major cardiovascular events as shown in the CANTOS outcome trial.^39^ In states of hTG, triglyceride-rich lipoproteins cross the endothelial barrier into the arterial wall and exert proinflammatory effects.^40–42^ Learning more about the residual inflammatory risk in hTG and factors associated with it would therefore be valuable when evaluating cardiovascular risk.^43^

Treatment of dyslipidaemia has, since the introduction of statins in the 1980s, mainly been focused on the lowering of cholesterol and LDL cholesterol levels to reduce cardiovascular risk. However, in the last decade, there has been a renaissance to specifically target plasma triglyceride reduction. Novel and exclusive medicines aimed to reduce apoC3 or ANGPTL molecules are effective for such a purpose. In the foreseeable future, when pharmacological treatment of hTG will become available on a broader indication, the conclusions acquired from the STRIREG study could help to define the most vulnerable hTG patients who should be given priority to new treatment. The recent implementation of the prospective Australian Hypertriglyceridemia Registry is another important contribution that will help shed light on cardiometabolic diseases associated with hTG.^44^ To help set research aims and priorities when evaluating the STRIREG dataset, the consensus of expert opinions will be taken into account.^45^

## Data Availability

The data that support the findings of this study are not publicly available. The study presented here has been subject to an application to an ethical board and approved for publication related to the specific aim of our research project. With reference to the European General Data Protection Regulation, the data are personal data and thereby protected by secrecy.

## Lead author biography

Dr Jonas Brinck is a senior consultant in Endocrinology at Karolinska University Hospital, Stockholm, Sweden. He completed his clinical training in internal medicine and endocrinology–diabetology in 2011 and pursued a post-doctoral fellowship in 2011–15 in Geneva, Switzerland, focusing on the protective effectiveness of lipoproteins in cardiac reperfusion injury. Dr Brinck is today an Associate Professor and group leader at the Department of Medicine Huddinge, Karolinska Institutet, Stockholm. His main research interest lies in the impact of hereditary traits familial hypercholesterolaemia and elevated lipoprotein(a) on cardiovascular health studied both in epidemiological and experimental contexts. Dr Brinck is a key opinion leader in the field of clinical dyslipidaemia in Sweden and Scandinavia.
